# A large scale multi institutional study for radiomics driven machine learning for meningioma grading

**DOI:** 10.1038/s41598-024-78311-8

**Published:** 2024-10-31

**Authors:** Mert Karabacak, Shiv Patil, Rui Feng, Raj K. Shrivastava, Konstantinos Margetis

**Affiliations:** 1 Department of Neurosurgery, Mount Sinai Health System, New York, NY USA; 2 Sidney Kimmel Medical College, Thomas Jefferson University, Philadelphia, PA USA

**Keywords:** Meningioma, Grading, Radiomics, Machine learning, MRI, Machine learning, Predictive medicine

## Abstract

This study aims to develop and evaluate radiomics-based machine learning (ML) models for predicting meningioma grades using multiparametric magnetic resonance imaging (MRI). The study utilized the BraTS-MEN dataset’s training split, including 698 patients (524 with grade 1 and 174 with grade 2–3 meningiomas). We extracted 4872 radiomic features from T1, T1 with contrast, T2, and FLAIR MRI sequences using PyRadiomics. LASSO regression reduced features to 176. The data was split into training (60%), validation (20%), and test (20%) sets. Five ML algorithms (TabPFN, XGBoost, LightGBM, CatBoost, and Random Forest) were employed to build models differentiating low-grade (grade 1) from high-grade (grade 2–3) meningiomas. Hyperparameter tuning was performed using Optuna, optimizing model-specific parameters and feature selection. The CatBoost model demonstrated the best performance, achieving an area under the receiver operating characteristic curve (AUROC) of 0.838 [95% confidence interval (CI): 0.689–0.935], precision of 0.492 (95% CI: 0.371–0.623), recall of 0.838 (95% CI: 0.689–0.935), F1 score of 0.620 (95% CI: 0.495–0.722), accuracy of 0.729 (95% CI: 0.650–0.800), an area under the precision-recall curve (AUPRC) of 0.620 (95% CI: 0.433–0.753), and Brier score of 0.156 (95% CI: 0.122-0.200). Other models showed comparable performance, with mean AUROCs ranging from 0.752 to 0.784. The radiomics-based ML approach presented in this study showcases the potential for non-invasive and pre-operative grading of meningiomas using multiparametric MRI. Further validation on larger and independent datasets is necessary to establish the robustness and generalizability of these findings.

**Supplementary Information:**

The online version contains supplementary material available at 10.1038/s41598-024-78311-8.

## Introduction

Meningiomas are the most common type of primary central nervous system neoplasm, comprising an estimated 39.7% of intracranial tumors^1^. The World Health Organization (WHO) classification system categorizes meningiomas into three grades based on their histological features: benign (grade 1), atypical (grade 2), and anaplastic (grade 3)^2^. Meningioma grades differ significantly in their prognosis and recurrence rates, with high-grade lesions (grades 2 and 3) generally exhibiting more aggressive behavior than low-grade lesions (grade 1), leading to poorer outcomes^3^. Most cases of meningioma are benign (80%), with a 5-year progression-free survival of 95.7%^4,5^. In contrast, the 5-year progression-free survival rates for grade 2 (18% of meningiomas) and grade 3 lesions (2% of meningiomas) are 81.8% and 46.7%, respectively^5^. Given this wide variation in meningioma progression, accurate tumor classification is critical for determining the optimal treatment approach, which can range from surveillance for low-grade lesions to surgical resection with adjuvant radiation for high-grade tumors^3,6^.

Magnetic resonance imaging (MRI) is considered the most appropriate neuroimaging modality for the initial diagnosis of meningiomas^7^. Research initiatives have focused on evaluating the pre-operative grading of meningiomas using MRI, potentially providing earlier and non-invasive insights into tumor characteristics. While several studies have reported that conventional features derived from MRI scans can discriminate between low-grade and high-grade meningiomas, there are notable inconsistencies in findings^8–10^. These inconsistencies may be attributed to the significant heterogeneity in radiographic features of tumors within the same grade, as well as the occurrence of overlapping features among lesions of different grades, posing a major challenge to accurate pre-operative and non-invasive meningioma grading^11^.

In recent years, advancements in radiomics and artificial intelligence (AI) have enabled the extraction of high-throughput radiographic features from imaging data that may not be perceived by the naked eye, such as tumor shape, texture, and intensity^12^. These radiomic features can reflect the underlying pathophysiology of lesions, particularly when combined with machine learning (ML) algorithms capable of correlating such imaging signatures with tumor characteristics^13^. Radiomics-based ML models can also incorporate information from multiparametric MRI (mpMRI) sequences, thereby providing a more comprehensive analysis of tumor grade. Indeed, several studies have demonstrated the ability of radiomics to classify meningioma grade using features extracted from mpMRI^14–16^.

However, the evidence from many of these studies is limited by small sample sizes or single-institutional cohorts, which can constrain the generalizability of radiomics-based models. Moreover, some studies have suggested that models utilizing mpMRI may perform sub-optimally compared to classifiers based on single-sequence MRI^10^. The performance of radiomics-based models is vulnerable to small sample sizes, which increase the risk of overfitting—a detriment particularly pertinent to multiparametric models that require extraction of a large number of features. To address these limitations, we aim to develop and evaluate five radiomics-based ML models for predicting meningioma grade using mpMRI data obtained from a large, multi-institutional cohort. To our knowledge, our study utilizes the largest sample size reported in the literature on radiomics-based meningioma grade prediction.

## Methods

### Data source and study population

This study utilized the BraTS-MEN dataset, a large multi-site, heterogeneous, multi-parametric MRI (mpMRI) meningioma dataset^17^. The dataset was contributed by academic medical centers across the United States. The BraTS-MEN dataset was originally created as part of a segmentation challenge aimed at developing and evaluating automated algorithms for delineating meningioma tumor boundaries on MRI scans. Cases were identified based on histopathologic assessment following resection or biopsy, or based on a clinical and radiographic diagnosis of meningioma.

For this study, we used the released training split of the BraTS-MEN dataset, as the segmentation masks for the validation split were not publicly available at the time this study was conducted, and developing an automatic segmentation model for meningiomas was beyond the scope of the current study. The initial dataset contained 1000 scans. For patients with multiple MRI scans at different time points, we used only the first available scan, reducing the dataset to 933 scans of 933 patients. We further excluded patients without grading information, resulting in a final dataset of 698 patients [524 (75.1%) with grade 1, 156 (22.3%) with grade 2, and 18 (2.6%) with grade 3 meningiomas].

Each case included pre-operative mpMRI consisting of pre-contrast T1-weighted (T1W), post-contrast T1-weighted (T1C), T2-weighted (T2W), and T2-weighted Fluid Attenuated Inversion Recovery (FLAIR) sequences. The dataset provided pre-processed images, including co-registered and skull-stripped volumes, as well as segmentation masks for three distinct tumor sub-regions: enhancing tumor, non-enhancing tumor core, and surrounding non-enhancing T2/FLAIR hyperintensity (SNFH). The details of the segmentation process are provided elsewhere^17^.

### Outcome of interest and study design

Our primary objective was to develop a non-invasive method for predicting meningioma grade using only pre-operative mpMRI scans. We approached this as a binary classification problem, aiming to distinguish between low-grade (grade 1) and high-grade (grade 2–3) meningiomas based solely on radiomic features extracted from the pre-operative mpMRI data. This approach could potentially aid in pre-operative planning and patient management by providing an early indication of tumor grade without requiring invasive procedures.

### Data splitting

We split the data into training (60%), validation (20%), and test (20%) sets. The training set was used for dimensionality reduction and model training. The validation set was used for hyperparameter optimization and model calibration. The test set remained completely independent throughout the entire model development process and was used solely for final model evaluation to obtain unbiased estimates of model performance.

### Feature extraction and dimensionality reduction

For feature extraction, we used a single binary segmentation mask obtained by merging the non-enhancing tumor core and enhancing tumor regions, representing the whole tumor without the SNFH. We extracted radiomic features from each patient’s mpMRI sequences (T1W, T1C, T2W, and FLAIR) using the PyRadiomics package^18^. A total of 4872 radiomic features were extracted for each patient.

The extraction process yielded eleven distinct categories of features, including original and wavelet-transformed features. The wavelet transformations included low-low-low (LLL), high-high-high (HHH), and various combinations (LLH, LHH, LHL, HLL, HHL, HLH), where ‘L’ denotes a low-pass filter and ‘H’ represents a high-pass filter. Each feature category encompassed five separate feature matrices: first-order statistics (18 features), Gray Level Co-occurrence Matrix (GLCM, 24 features), Gray Level Size Zone Matrix (GLSZM, 16 features), Gray Level Run Length Matrix (GLRLM, 16 features), and Gray Level Dependence Matrix (GLDM, 14 features). An additional shape matrix (14 features) was included within the original feature category. These features capture a diverse range of information from the image data, including intensity distributions, textures, shapes, and wavelet transforms, enhancing the comprehensiveness of the data analyzed by our ML models.

To reduce the dimensionality of the feature space and mitigate the risk of overfitting, we employed LASSO (Least Absolute Shrinkage and Selection Operator) regression. The LASSO model uses the tuning parameter ‘alpha’ parameter for feature selection. As alpha decreases, some of the model’s coefficients are reduced to zero. We set alpha to 0.001, which resulted in most coefficients being reduced to zero, leaving only the most predictive features. This technique reduced the number of features to 176, which were then sorted in descending order of importance for subsequent use in model development. This feature selection process was performed using only the training set. The validation set was used for hyperparameter optimization and model calibration, while the test set remained completely independent throughout the entire process, reserved solely for final model evaluation.

All analyses in this study were performed using Google Colab with a CPU runtime. This cloud-based platform was sufficient for running our machine learning models, including feature extraction, dimensionality reduction, and model training processes. The use of traditional machine learning algorithms, as opposed to more computationally intensive deep learning models, allowed us to complete our analyses without the need for specialized hardware or GPU acceleration. This approach demonstrates the computational efficiency of our methodology.

### Model development and evaluation

Our study employed five different supervised ML algorithms to construct predictive models: TabPFN, XGBoost, LightGBM, CatBoost, and Random Forest. Each algorithm was used to build a separate model for meningioma grade prediction. To address the class imbalance in the training set, we applied the Synthetic Minority Over-sampling Technique (SMOTE). SMOTE creates synthetic examples of the minority class (in this case, high-grade meningiomas) by interpolating between existing minority class instances, thereby balancing the dataset^19^.

Feature scaling was performed using min-max scaling, which scales all numeric variables to a fixed range between 0 and 1. This scaling helps to standardize the range of independent variables and can improve the performance and training stability of many ML algorithms. The scaler was fit on the training data and then applied to the validation and test sets.

We used Optuna, a hyperparameter optimization framework^20^, to tune the hyperparameters for each model, with the goal of maximizing the area under the receiver operating characteristic curve (AUROC) as the optimization metric. The optimization process involved 100 trials for each model, determining both the optimal model-specific parameters and the number of features to be used.

During this optimization process, feature selection was performed dynamically. For each trial, the number of top features to use was treated as a hyperparameter, allowing the model to select an optimal subset from the previously sorted list of features. This approach enabled each model to determine its ideal balance between including informative features and avoiding overfitting.

For each model, we performed hyperparameter optimization using Optuna, selected features based on the optimal number determined during optimization, fit the model on the training data, and calibrated the model using sigmoid calibration^21^. Following model development, we evaluated the final performance exclusively on the held-out test set, which had not been used in any way during model development or tuning.

We evaluated model performance using several metrics, with the AUROC as the primary metric. Additional metrics included precision, recall, F1 score, accuracy, Matthews Correlation Coefficient (MCC), the area under the precision-recall curve (AUPRC), and Brier score. We calculated 95% confidence intervals (CIs) for these metrics using bootstrap resampling with 1000 iterations. For binary classification metrics (precision, recall, F1, accuracy, MCC), we utilized the Youden index to determine the optimal classification threshold^22,23^. We identified this threshold as the point on the receiver operating characteristic (ROC) curve corresponding to the maximum value of the Youden Index (J = sensitivity + specificity – 1), which is a common metric in diagnostic or prognostic test evaluation.

To aid in model interpretation and visualization, we generated ROC curves, precision-recall curves (PRCs), calibration curves, and confusion matrices. We also computed SHAP (SHapley Additive exPlanations) values to provide insights into feature importance and model decision-making processes^24^.

## Results

Our final dataset consisted of 698 patients with a mean age of 58.9 years, including 476 females and 222 males. Of these, 524 patients had low-grade (grade 1) meningiomas (75.1%), and 174 (24.9%) had high-grade (grade 2–3) meningiomas.

The hyperparameter tuning process resulted in different optimal numbers of features for each model: TabPFN used 56 features, XGBoost used 162, LightGBM used 150, CatBoost used 134, and Random Forest used 140. The optimal binary classification thresholds, determined using the Youden index, were as follows: TabPFN (20.2%), XGBoost (20.8%), LightGBM (17.2%), CatBoost (11.7%), and Random Forest (18.6%).

All five ML models demonstrated performance superior to random chance in predicting meningioma grade based on radiomic features extracted from multiparametric MRI (Table 1). The CatBoost model achieved the highest overall performance, with a mean AUROC of 0.838 (95% CI: 0.689–0.935). It also demonstrated the highest recall of 0.838 (95% CI: 0.689–0.935) and F1 score of 0.620 (95% CI: 0.495–0.722) among all models. The performance metrics for the CatBoost model were as follows: precision 0.492 (95% CI: 0.371–0.623), accuracy 0.729 (95% CI: 0.650–0.800), MCC 0.467 (95% CI: 0.317–0.597), AUPRC 0.620 (95% CI: 0.433–0.753), and Brier score 0.156 (95% CI: 0.122-0.200).

**Table 1:** Performance metrics of the models (MCC, Matthews Correlation Coefficient; AUROC, the area under the receiver operating characteristic curve; AUPRC, the area under the precision-recall curve).

	TabPFN	XGBoost	LightGBM	CatBoost	Random Forest
Number of selected features	56	162	150	134	140
Optimal classification threshold	20.20%	20.80%	17.20%	11.70%	18.60%
Precision (95% CI)	**0.543** **(0.400–0.682)**	0.490 (0.355–0.642)	0.481 (0.349–0.632)	0.492 (0.371–0.623)	0.474 (0.352–0.618)
Recall (95% CI)	0.676 (0.514–0.806)	0.649 (0.479–0.783)	0.676 (0.512–0.806)	**0.838** **(0.689–0.935)**	0.730 (0.577–0.854)
F1 score (95% CI)	0.602 (0.467–0.722)	0.558 (0.434–0.681)	0.562 (0.435–0.682)	**0.620** **(0.495–0.722)**	0.574 (0.456–0.701)
Accuracy (95% CI)	**0.764** **(0.686–0.829)**	0.729 (0.657-0.800)	0.721 (0.643–0.793)	0.729 (0.650–0.800)	0.714 (0.636–0.786)
MCC (95% CI)	0.443 (0.264–0.586)	0.375 (0.201–0.533)	0.377 (0.211–0.531)	**0.467** **(0.317–0.597)**	0.394 (0.229–0.536)
AUROC (95% CI)	0.752 (0.641–0.840)	0.769 (0.674–0.846)	0.784 (0.693–0.856)	**0.811** **(0.716–0.875)**	0.782 (0.679–0.858)
AUPRC (95% CI)	0.567 (0.401–0.711)	0.553 (0.373–0.702)	0.610 (0.448–0.737)	0.620 (0.433–0.753)	**0.625** **(0.447–0.764)**
Brier score (95% CI)	0.160 (0.123–0.203)	0.163 (0.122–0.214)	0.163 (0.124–0.212)	**0.156** **(0.122-0.200)**	0.155 (0.118–0.203)

The LightGBM and Random Forest models also performed well, with mean AUROCs of 0.784 (95% CI: 0.693–0.856) and 0.782 (95% CI: 0.679–0.858), respectively. The XGBoost and TabPFN models showed slightly lower performance, with mean AUROCs of 0.769 (95% CI: 0.674–0.846) and 0.752 (95% CI: 0.641–0.840), respectively.

Notably, the confidence intervals for most metrics overlapped across models, suggesting that the differences in performance may not be statistically significant in all cases. However, the consistent trend of CatBoost outperforming other models across multiple metrics indicates its potential as a robust tool for meningioma grade prediction.

**Fig. 1:**
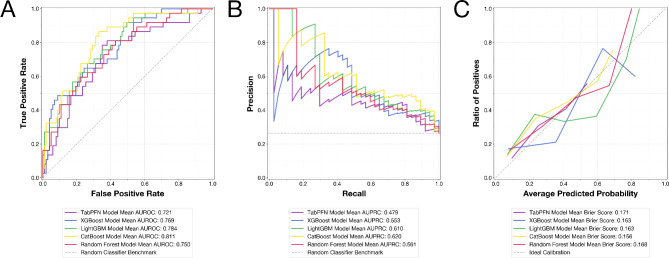
Models’ (**A**) receiver operating characteristics curves, (**B**) precision-recall curves, and (**C**) calibration curves (AUROC: area under receiver operating characteristics curve; AUPRC: area under receiver operating characteristics curve).

**Fig. 2:**
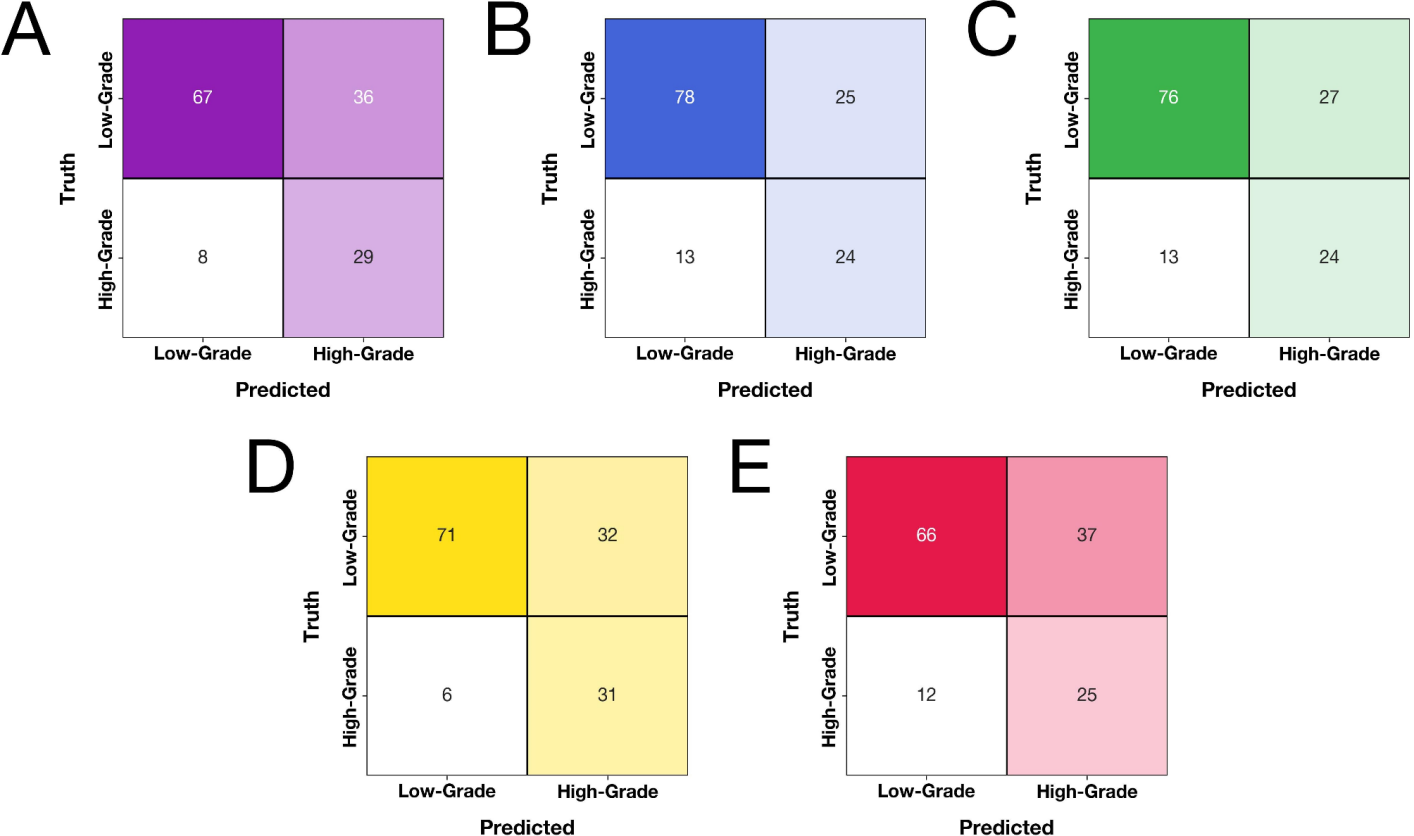
Confusion matrices of the models built with the (**A**) TabPFN, (**B**) XGBoost, (**C**) LightGBM, (**D**) TabPFN, and (**E**) Random Forest algorithms.

The ROC curves (Fig. 1A) and PRCs (Fig. 1B) visually confirmed the strong performance of the models, with the CatBoost model consistently demonstrating superior curves compared to other algorithms. Calibration curves showed that all models were reasonably well-calibrated, with CatBoost exhibiting the best calibration (Fig. 1C). Confusion matrices provided a detailed breakdown of true positive, true negative, false positive, and false negative predictions for each model, further illustrating CatBoost’s superior performance across various classification scenarios (Fig. 2). SHAP analysis revealed the most important features for predicting meningioma grade across the different models (Online Resource 1–5).

## Discussion

In this study, we evaluated the performance of five radiomics-based ML models in predicting meningioma grade using features extracted from T1W, T1C, T2W, and FLAIR MRI sequences. All models demonstrated fair to good discriminative abilities^25^, with the CatBoost model achieving the highest overall performance: a mean AUROC of 0.838 (95% CI: 0.689–0.935), recall of 0.838 (95% CI: 0.689–0.935), and F1 score of 0.620 (95% CI: 0.495–0.722). Across all models, significant features for meningioma grade prediction were derived from all MRI modalities used in the study. The most important feature for most models was the ‘T1C GLSZM small area emphasis’, except for the CatBoost model, for which ‘T2 wavelet LLH GLCM joint energy’ was found to be the most important feature. GLSZM and GLCM are measures of image texture that capture differences in pixel intensities and spatial relationships. These features may correlate with tumor cellularity and, consequently, tumor grade^26,27^.

The superior performance of the CatBoost model in our study is particularly noteworthy given that our dataset consisted entirely of numerical features, despite CatBoost’s design strength in handling categorical data^28^. This suggests that CatBoost’s advantages extend beyond its categorical feature handling. Key factors contributing to its performance likely include its gradient boosting framework, which excels at capturing complex, non-linear relationships in high-dimensional data^28^. CatBoost’s unique ordered boosting approach helps reduce prediction shift, a common issue in gradient boosting methods. Its symmetric tree structure and feature combinations generation allow for effective modeling of intricate patterns in radiomics features. Moreover, CatBoost’s built-in mechanisms for handling overfitting, such as ordered boosting and feature randomization, likely contributed to its robust performance on our validation and test sets.

Our study analyzed a sample of 698 patients (524 low-grade, 174 high-grade), representing the largest cohort to date in the literature on radiomics-based meningioma grade prediction. The use of a multi-institutional dataset significantly enhances the generalizability of our findings, addressing a common limitation in radiomics studies. Models trained on data from a single institution may lead to overfitting, achieving high accuracy on internal validation datasets but performing poorly when tested on external cohorts. By incorporating data from multiple institutions, our study intentionally includes variability in imaging protocols, scanner vendors, and image quality. This diversity in our dataset is a key strength, as it reflects the real-world heterogeneity encountered in clinical settings. Our data splitting strategy ensured that this variability was represented across our training, validation, and test sets, allowing us to develop and evaluate models that are robust to differences in imaging parameters. The strong performance of our models, particularly CatBoost, in this heterogeneous setting underscores their potential for broad clinical applicability. Training models with such diverse imaging data is crucial to account for real-world variability in image acquisition and to develop tools that can generalize effectively across different clinical environments^26^. Furthermore, our models integrated features extracted from mpMRI, contributing to the high accuracy achieved. Each MRI sequence provides unique information about the tumor, which, when combined, allows for a comprehensive characterization of the meningioma. For instance, T1C is considered to reveal tumor heterogeneity based on the distribution of contrast agents in the extracellular space^29^. Non-contrast sequences, on the other hand, may reflect tumor characteristics that are potentially masked by contrast enhancement, such as uniformity, texture depth, and degree of thickness^10,11^.

Several studies have evaluated the performance of radiomics-based ML models in predicting meningioma grade, providing context for our findings. Hu et al. achieved higher accuracy with a fusion model (AUROC 0.840) compared to a single-sequence MRI model (AUROC 0.77), although their study lacked validation by an external control group^10^. Ke et al. demonstrated the superiority of a multiparametric model (T1 + T2 + T1C, AUROC 0.830) over single-sequence models, and enhanced the validity of their results by incorporating an external validation set^15^. However, their study included only 184 patients from two institutions. Hamerla et al. reported a very high accuracy (AUROC 0.970) with their multiparametric model, but the analysis included only 48 out of 138 cases, suggesting a potential risk of overfitting^29^. Our study builds upon and extends these previous efforts in several important ways. First, we utilized a substantially larger dataset of 698 patients, which is the largest cohort to date for radiomics-based meningioma grade prediction. This larger sample size enhances the statistical power and reliability of our findings. Second, our data were collected from multiple institutions across the United States, incorporating a wider range of imaging protocols and patient populations. This multi-institutional approach significantly improves the generalizability of our models compared to single-center studies. Third, we employed a rigorous validation strategy, using not only a validation set but also a separate test set, which provides a more robust evaluation of model performance on truly unseen data. This is in contrast to studies like Hamerla et al. that relied solely on cross-validation^29^. Importantly, our study goes beyond merely reporting discrimination metrics like AUROC. We also provide calibration curves and the Brier score, a calibration metric. This focus on calibration addresses what has been termed ‘the Achilles heel of predictive analytics’^30^. Well-calibrated models are crucial for reliable risk prediction in clinical settings, as they ensure that predicted probabilities closely match observed outcomes. By reporting these metrics, our study offers a more comprehensive assessment of model performance that is critical for potential clinical application.

Furthermore, we have made our code publicly available, enabling other researchers to externally validate and build upon our approach. This transparency and reproducibility are critical for advancing the field and facilitating clinical translation. While the models evaluated in our study demonstrated accuracy and performance metrics comparable to those reported in the literature^31,32^, the combination of a larger sample size, multi-institutional data, rigorous validation, comprehensive performance metrics including calibration, and code availability provides a more complete and potentially more clinically applicable approach to radiomics-based meningioma grading.

Meningioma grade is a crucial determinant of survival prognosis and treatment strategy. Currently, the gold standard for tumor grading relies on surgical resection and subsequent histopathological analysis^2^. However, the ability to accurately differentiate between low- and high-grade tumors using pre-operative imaging could significantly improve clinical care. This non-invasive approach could aid in early treatment planning in several ways. For tumors in challenging locations, pre-operative grade prediction could help weigh the risks and benefits of aggressive resection versus more conservative management. In cases where immediate intervention isn’t necessary, the predicted grade could inform the frequency and intensity of follow-up imaging. More accurate pre-operative grading could enhance discussions with patients about prognosis and treatment options. Clinicians could better plan for the potential need for adjuvant treatments, especially for predicted high-grade tumors. Additionally, pre-operative grading could aid in patient stratification for clinical trials of new therapies. Our radiomics-based approach provides valuable additional information for characterizing tumors and predicting their behavior, potentially influencing the timing and approach of interventions. These findings contribute to a growing body of evidence supporting the potential application of radiomics-based machine learning models in this critical domain of neuro-oncology, offering a complementary tool to enhance current clinical decision-making processes.

An important consideration in interpreting our results is the clinical application of our model’s predictions. While our model demonstrates high sensitivity (83.8%) for detecting high-grade meningiomas, its positive predictive value of 49.2% means that when the model predicts a high-grade tumor, the patient still has a slightly higher chance of having a low-grade tumor. This characteristic requires careful consideration in clinical implementation. Rather than serving as a definitive diagnostic tool, we envision this model as a risk stratification tool to inform clinical decision-making. The model’s strength lies in its ability to reliably identify low-grade tumors (high negative predictive value) while flagging cases that may require additional attention. For patients with a ‘high-grade’ prediction, this result could inform management strategies such as more frequent imaging follow-up, consideration of additional imaging sequences, earlier surgical consultation, or more detailed discussion of treatment options. This approach leverages the model’s high sensitivity for detecting aggressive tumors while acknowledging the need to interpret positive predictions in the context of other clinical and radiological findings.

Our study has several limitations that warrant consideration. First, the retrospective design introduces a risk of selection bias. Future research should address this by implementing prospective study designs. Second, the distribution of tumor grades in our dataset was uneven, reflecting the relatively low incidence of high-grade lesions in real-world scenarios. To address this, we employed the SMOTE technique to balance class distributions in our training set. However, it’s important to note that SMOTE was only applied to the training data; our validation and test sets maintained their original, unbalanced distributions. This approach allows us to develop more balanced models while still evaluating their performance on data that reflects real-world grade distributions. Although this methodology aims to minimize bias, we acknowledge that there may still be a possibility of residual bias towards the majority class (low-grade meningiomas). Our strong performance metrics on the unaltered validation and test sets suggest that our models can effectively handle uneven grade distributions, but further validation in diverse clinical settings would be beneficial to fully assess the impact of this approach on real-world application. Third, we could not incorporate diffusion-weighted imaging (DWI) or apparent diffusion coefficient (ADC) maps into our models due to their unavailability in the BraTS-MEN dataset. DWI and ADC may reflect distinct characteristics of meningiomas that could complement radiomics features extracted from conventional MRI. For instance, ADC values have been shown to correlate inversely with the Ki-67 index and cellularity^33^. ncluding these quantitative indicators might further improve the performance of radiomics-based models in predicting tumor grade. However, this would require an even larger sample size to account for the increase in extracted features. Moreover, obtaining a sufficiently large sample with DWI and ADC may be challenging, as not all pre-operative evaluations of meningioma include these sequences^29^. Finally, while we used a large multi-institutional cohort and employed rigorous validation techniques that approximate external validation, we did not validate our models with a completely independent external control group. However, we believe our approach provides a strong foundation for generalizability. We encourage future research to further validate and build upon our findings by applying our publicly available code to new, independent datasets. This will provide additional insights into the generalizability and clinical applicability of radiomics-based machine learning models for meningioma grading.

## Conclusion

This study developed and evaluated five radiomics-based ML models to differentiate between low-grade and high-grade meningiomas using pre-operative mpMRI. By leveraging a large, multi-institutional cohort, our study addresses key limitations present in previous research on this topic. The promising performance of our models, particularly the CatBoost algorithm, demonstrates the potential of radiomics-based ML models to non-invasively determine meningioma grades. This approach could significantly advance the management of meningioma patients by enabling more informed decision-making prior to invasive procedures. Our findings contribute to the growing body of evidence supporting the integration of AI in neuro-oncology, paving the way for more personalized and efficient patient care. Future studies should focus on external validation and prospective evaluation to further establish the clinical utility of these models.

## Electronic supplementary material

Below is the link to the electronic supplementary material.

Supplementary Material 1

Supplementary Material 2

Supplementary Material 3

Supplementary Material 4

Supplementary Material 5

## Data Availability

The BraTS-MEN data are publicly available on Synapse [Calabrese, E. & LaBella, D. BraTS Meningioma Dataset. Synapse https://doi.org/10.7303/syn51514106].
