# Supplementary material for: A large scale multi institutional study for radiomics driven machine learning for meningioma grading
Source: Sci Rep. 2024 Oct 31;14:26191. doi: 10.1038/s41598-024-78311-8 (PMC11525589; doi:10.1038/s41598-024-78311-8)

**Online Resource 4.** The 19 most important features, along with the sum of the remaining 115 features and their mean SHAP values for the models built with the CatBoost algorithm.


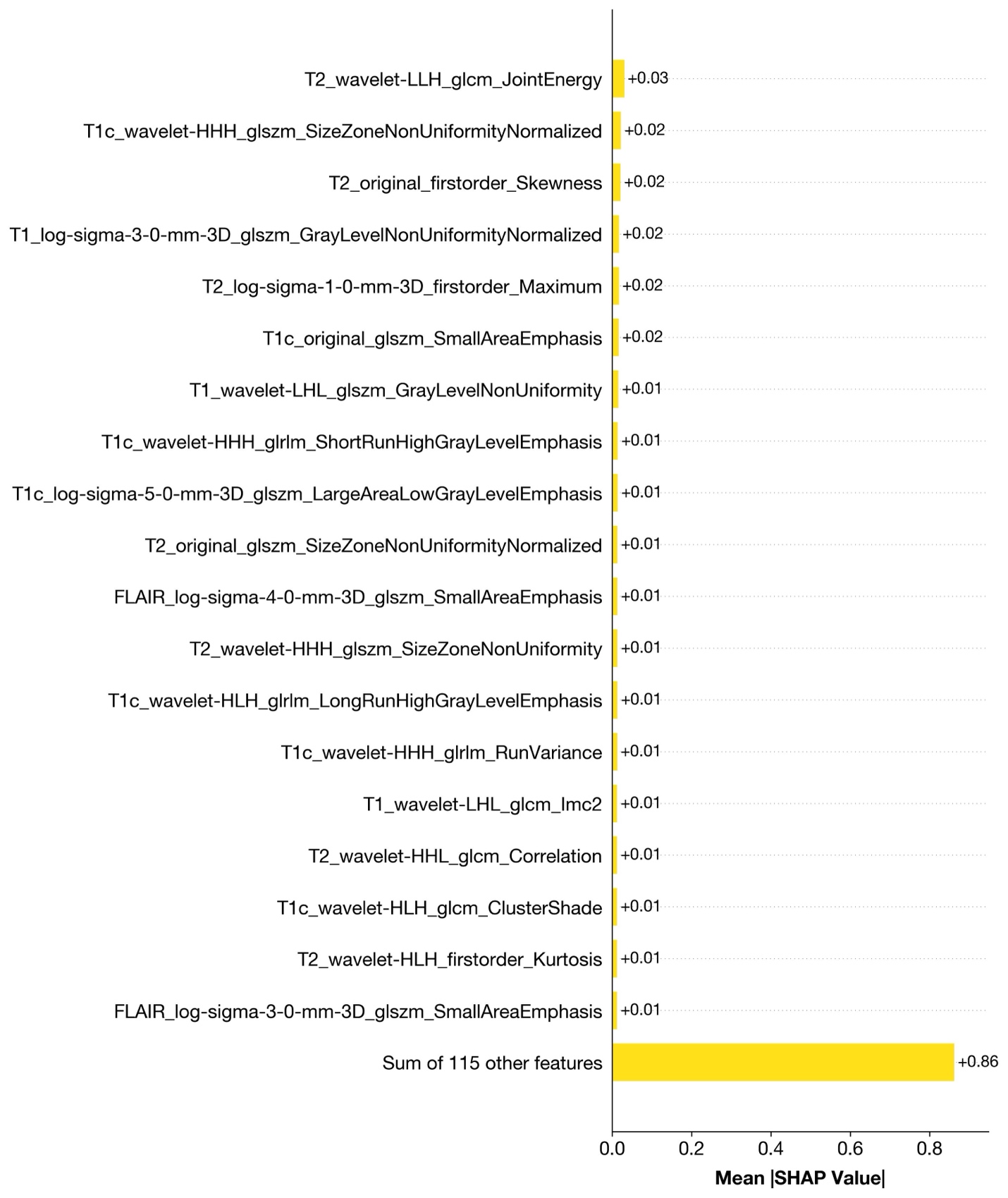

Supplement: Supplementary file 4 — Supplementary Material 4 [file 41598_2024_78311_MOESM4_ESM.docx]
